# Metabolic control analysis of respiration in human cancer tissue

**DOI:** 10.3389/fphys.2013.00151

**Published:** 2013-06-28

**Authors:** Tuuli Kaambre, Vladimir Chekulayev, Igor Shevchuk, Kersti Tepp, Natalja Timohhina, Minna Varikmaa, Rafaela Bagur, Aleksandr Klepinin, Tiia Anmann, Andre Koit, Andrus Kaldma, Rita Guzun, Vahur Valvere, Valdur Saks

**Affiliations:** ^1^Laboratory of Bioenergetics, National Institute of Chemical Physics and BiophysicsTallinn, Estonia; ^2^Laboratory of Fundamental and Applied Bioenergetics, Joseph Fourier UniversityGrenoble, France; ^3^Oncology and Hematology Clinic at the North Estonia Medical CentreTallinn, Estonia; ^4^Competence Centre for Cancer ResearchTallinn, Estonia

**Keywords:** metabolic control analysis, respiratory chain, breast and colorectal cancer, Warburg effect, OXPHOS

## Abstract

Bioenergetic profiling of cancer cells is of great potential because it can bring forward new and effective therapeutic strategies along with early diagnosis. Metabolic Control Analysis (MCA) is a methodology that enables quantification of the flux control exerted by different enzymatic steps in a metabolic network thus assessing their contribution to the system‘s function. Our main goal is to demonstrate the applicability of MCA for *in situ* studies of energy metabolism in human breast and colorectal cancer cells as well as in normal tissues. We seek to determine the metabolic conditions leading to energy flux redirection in cancer cells. A main result obtained is that the adenine nucleotide translocator exhibits the highest control of respiration in human breast cancer thus becoming a prospective therapeutic target. Additionally, we present evidence suggesting the existence of mitochondrial respiratory supercomplexes that may represent a way by which cancer cells avoid apoptosis. The data obtained show that MCA applied *in situ* can be insightful in cancer cell energetic research.

## Introduction

Oncologic diseases such as breast and colorectal cancers are still one of the main causes of premature death among people. The low efficiency of contemporary medicine in the treatment of these malignancies is largely mediated by a poor understanding of the processes involved in metastatic dissemination of cancer cells as well as the unique energetic properties of mitochondria from tumors. Current knowledge supports the idea that human breast and colorectal cancer cells exhibit increased rates of glucose consumption displaying a Warburg phenotype, i.e., elevated glycolysis even in the presence of oxygen (Warburg and Dickens, 1930; Warburg, 1956; Izuishi et al., 2012). Notwithstanding, there are some evidences that in these malignancies mitochondrial oxidative phosphorylation (OXPHOS) is the main source of ATP rather than glycolysis. Cancer cells have been classified according to their pattern of metabolic remodeling depending of the relative balance between aerobic glycolysis and OXPHOS (Bellance et al., 2012). The first type of tumor cells is highly glycolytic, the second OXPHOS deficient and the third type of tumors display enhanced OXPHOS. Recent studies strongly suggest that cancer cells can utilize lactate, free fatty acids, ketone bodies, butyrate and glutamine as key respiratory substrates eliciting metabolic remodeling of normal surrounding cells toward aerobic glycolysis—“reverse Warburg” effect (Whitaker-Menezes et al., 2011; Salem et al., 2012; Sotgia et al., 2012; Witkiewicz et al., 2012). In normal cells, the OXPHOS system is usually closely linked to phosphotransfer systems, including various creatine kinase (CK) isotypes, which ensure a safe operation of energetics over a broad functional range of cellular activities (Dzeja and Terzic, 2003). However, our current knowledge about the function of CK/creatine (Cr) system in human breast and colorectal cancer is insufficient. In some malignancies, for example sarcomas the CK/Cr system was shown to be strongly down regulated (Bera et al., 2008; Patra et al., 2008). Our previous studies showed that the mitochondrial-bound CK (MtCK) activity was significantly decreased in HL-1 tumor cells (Monge et al., 2009), as compared to normal parent cardiac cells where the OXPHOS is the main ATP source of and the CK system is a main energy carrier. In the present study, we estimated the role of MtCK in maintaining energy homeostasis in human colorectal cancer cells.

Understanding the control and regulation of energy metabolism requires analytical tools that take into account the existing interactions between individual network components and their impact on systemic network function. Metabolic Control Analysis (MCA) is a theoretical framework relating the properties of metabolic systems to the kinetic characteristics of their individual enzymatic components (Fell, 2005). An experimental approach of MCA has been already successfully applied to the studies of OXPHOS in isolated mitochondria (Tager et al., 1983; Kunz et al., 1999; Rossignol et al., 2000) and in skinned muscle fibers (Kuznetsov et al., 1997; Tepp et al., 2010).

Recent work from Moreno-Sanchez and Westerhoff's groups has applied MCA to investigate the control of glycolytic flux and mitochondrial respiration in different types of tumor cells growing in culture. A main conclusion of these studies is that the significance of OXPHOS in bioenergetics of cancer cells should be re-evaluated and experimentally determined for each particular type of neoplasm (Marin-Hernandez et al., 2006; Moreno-Sanchez et al., 2007, 2008, 2010). These findings also indicated that MCA may be a very useful approach for studying *in situ* mitochondrial respiration and energy fluxes.

In the present work we applied MCA for *in situ* studies the energy metabolism in human cancer cells. Using oxygraphy and MCA in permeabilized human breast and colorectal cancer cells (Kuznetsov et al., 1997) we quantitatively characterized the control exerted by the different components of the respiratory chain and the ATP synthasome (Tepp et al., 2011).

## Materials and methods

### Patients and tissue sampling

Bioenergetic proiling was performed on post-operation material derived from patients with human breast (HBC) and colorectal cancers (HCC). Thirty two patients 50–71 year-old were in the HBC group with local or locally advanced disease at pathological stage IA-IIIB (T1-4N0-2M0) and eighteen patients, 63–82 year-old in HCC group with pathological stage (T2-3 N1-M0). Tumor differentiation was into well, moderately, and poorly differentiated adenocarcinoma.

Immediately after the surgery the human samples were placed into pre-cooled Mitomedium-B solution, dissected into small fiber bundles and permeabilized with 50 μg/ml saponin (Kuznetsov et al., 2008). Control experiments showed that this procedure has no effect on the integrity of mitochondrial membranes and that the stimulatory effect on respiration by added cytochrome-c is absent (Kuznetsov et al., 2008; Kaambre et al., 2012).

### High-resolution respirometry

Mitochondrial respiration of tissue samples was measured at 25°C under continuous magnetic stirring with an Oxygraph-2 k, (Oroboros Instruments, Innsbruck, Austria) 5 mM glutamate, 2 mM malate and 10 mM succinate were used as respiratory substrates. In permeabilized tumor and muscle fibers, the mitochondrial respiration was activated by exogenously added ADP. The flux control coefficients (FCC) for permeabilized human samples were determined with direct activation of respiration by ADP (state 3 respiration). The presence of MtCK in permeabilized human cancer samples was assayed as described earlier (Monge et al., 2009; Kaambre et al., 2012).

### Metabolic control analysis

By applying the principles of MCA, it is possible to quantify the degree of the control, exerted by an enzymatic or transport step through FCC. FCC is defined as the ratio of the fractional change in the steady-state flux with respect to an infinitesimal variation in the biochemical activity that caused the change in flux (Fell, 1997). In the present study FCC was assessed by stepwise titration of the respiratory activity of the system with the specific inhibitors for each step from the respiratory chain and ATP synthasome complexes. Control coefficients are determined from the initial slope of the titration curve and the ratio of inhibitor concentration at maximal flux inhibition over the uninhibited flux.

FCC-values were quantified according to a graphical method (Groen et al., 1982; Fell, 2005) modified by Small (Small and Fell, 1990) and results obtained were compared with the computer estimated coefficients (Gellerich et al., 1990; Small and Fell, 1990). Previous studies indicated that similar values can be obtained with either methods, but special attention should be paid to systems with branched pathways or direct channeling due to possible unreliable estimates (Kholodenko et al., 1993; Kholodenko and Westerhoff, 1993; Tepp et al., 2011).

## Results and discussion

### Bioenergetic profiling of human cancer and MCA

First, we evaluated the impact of Cr, ADP, mitochondrial-bound hexokinase (HK) and CK reactions on OXPHOS in permeabilized human tumor samples. It has been proposed that in cancer cells the binding of HK-2 to the mitochondrial voltage-dependent anion channel (VDAC) mediates their Warburg behavior further suggesting that this enzyme could be used as a target for antineoplastic therapy (Pedersen, 2008). We found that affecting mitochondria-bound CK and HK only produces minor effects on mitochondrial respiration in HCC cells. Indeed, the addition of 10 mM Cr and 5 mM glucose with 0.2 mM ATP (in the presence of phosphoenolpyruvate-pyruvate kinase ADP trapping system) had no effect on the rates of oxygen consumption by these cells. Similar effects were also registered in HBC cells (Kaambre et al., 2012). From these results, it appears that MtCK does not play a significant role in HBC and HCC cells *in situ*. The role of another CK isoforms in maintaining of energy homeostasis in these cancer cells will be examined in future work. The data obtained suggest that mitochondrial, but not glycolytic ATP, plays a key role in maintaining life processes in HCC and HBC cells. In contrast to HCC cells, a marked stimulatory effect on mitochondrial respiration by glucose addition (in the presence of exogenous MgATP) was observed in saponized HL-1 tumor cells that display a glycolytic phenotype (Eimre et al., 2008; Monge et al., 2009). Furthermore, we found that adding respiratory substrates and 2 mM ADP to HCC and HBC fibers resulted in a notable increase in O2 consumption rate (Table 1).

**Table 1:** **Values of basal (*V*_*o*_) and maximal respiration rate (*V*_max_, in the presence of 2 mM ADP) and apparent Michaelis Menten constant (*K*_*m*_) for ADP in permeabilized human breast and colorectal cancer samples as well as health tissue**.

**Tissues**	***V*_**o**_^#^**	***V_**o**_(succ)***	***K_**m**_^**app**^ADP*, μ*M***	***V*_max_**	**RCI**	**Source**
Colorectal cancer	1.4 ± 0.21	2.62 ± 0.34^*^	34.2 ± 11.1	4.51 ± 0.47^*^	3.2 ± 0.8	Our data
Control tissue	1.19 ± 0.17	1.61 ± 0.24	46.3 ± 15.5	2.56 ± 0.32	2.2 ± 0.6	Our data
Breast cancer	0.33 ± 0.03	0.56 ± 0.04	114.8 ± 13.6	1.09 ± 0.04	3.3 ± 0.4	Kaambre et al., 2012
Control breast tissue	0.02 ± 0.01	0.10 ± 0.02	–	–	–	Kaambre et al., 2012
Rat soleus	2.19 ± 0.30	–	354 ± 46	12.2 ± 0.5	5.6 ± 1.0	Kuznetsov et al., 1996; Monge et al., 2009
Rat gastrocnemius white	1.23 ± 0.13	–	14.4 ± 2.6	4.10 ± 0.25	3.3 ± 0.6	Kuznetsov et al., 1996; Monge et al., 2009

# Respiration rate is expressed in nmol O_2_/min/mg dry weight; V_o_—in the presence of 2 mM malate and 5 mM glutamate as respiratory substrates; V_o_(succ)—in the presence of 2 mM malate, 5 mM glutamate, and 10 mM succinate; RCI—respiratory control index is the ratio of V_max_ value to V_o_;

* p < 0.05 as compared to control tissue; data are expressed as mean ± standard error of the mean(SEM).

Due to the absence of MtCK in HCC cells, we further analyzed OXPHOS in these cells upon direct activation of respiration with exogenous ADP. In order to evaluate the functionality of individual respiratory complexes of the electron transport chain (ETC) in HCC and HBC cells, the rates of O_2_ consumption were measured after sequential addition of specific substrates and inhibitors in the following order: 2 mM ADP, 10 μM rotenone, 10 mM succinate, 10 μM antimycin A, 5 mM ascorbate with 1 mM tetramethyl-p-phenylenediamine (TMPD). We found that the addition of 2 mM ADP activates mitochondrial respiration by ~3.2 and 3.3 times in HCC and HBC samples, respectively (Table 1). Our studies showed that these human malignancies have a functionally active Krebs cycle as well as the ETC. Accordingly ADP stimulated respiration of human breast and colorectal tumors was found to be strongly depressed upon addition of 10 μM rotenone (an inhibitor of Complex-I), antimycin (an inhibitor of Complex-III), 1 mM NaCN (an inhibitor of Complex-IV) and, on the contrary, it was strongly (>5 times) activated in the presence of ascorbate with TMPD, indicating the presence of active cytochrome-c-oxidase (data not shown). Apparently, the activity of Complex-II in HCC exceeds that in normal tissue, as the addition of succinate to permeabilized fibers led to a stronger stimulation of respiration than in control tissue, although it has been reported that SDHD gene expression is reduced in ~80% colorectal cancers (Habano et al., 2003).

The results shown in Table 1 demonstrate that the respiratory activity of breast and colorectal cancers differ significantly that of normal adjacent tissues. Both tumors exhibited respiratory rates close to tissue from rat skeletal muscles (Table 1). These data may indicate the presence of a “reverse Warburg” effect, which depends on the properties of the tumor microenvironment. The microenvironment (e.g., substrate availability) is a strong determinant of mitochondrial content and activity in tumors, which could play an important role in the definition of tumors bioenergetic profile (Bellance et al., 2012; Jose and Rossignol, 2013).

When we analyzed respiration as a function of exogenously added ADP, we found that mitochondria from human breast and colorectal cancer cells exhibit an increased affinity toward exogenously added ADP compared with normal oxidative type tissues. The apparent Michaelis Menten constants (*K*_*m*_) for MgADP were determined as 114.8 ± 13.6 μM and 34.2 ± 11.1 μM for breast and colorectal cancer, respectively (Table 1). These values are significantly lower as compared to rat soleus (*K*_*m*_ = 354±46 μM) or isolated cardiomyocytes [*K*_*m*_ = 360 μM (Anmann et al., 2006)], but this value is still higher than the apparent *K*_*m*_ for isolated mitochondria (10–20 μM). The observed difference in the metabolic regulation of respiration could be linked to a decreased expression or absence of some cytoskeletal proteins (Appaix et al., 2003; Saks et al., 2010; Guzun et al., 2012). It has been shown that in normal oxidative muscle, βII-tubulin can bind to VDAC and thereby strongly limit the permeability of mitochondrial outer membrane to adenine nucleotides (Rostovtseva et al., 2008; Guzun et al., 2011). In addition to βII-tubulin, candidate proteins are desmin, microtubule-associated proteins, other isoforms of tubulin and plectin (Appaix et al., 2003; Guzun et al., 2012).

We used MCA to quantitate the control exerted by the different ETC complexes and the ATP synthasome on the respiratory flux in human colorectal and breast carcinomas. FCCs were determined in permeabilized human fibers using the inhibitor titration method in ADP-stimulated respiration.

In HBC cells, the main rate-controlling steps of respiration were Complex IV (*FCC* = 0.74), ATP synthase (*FCC* = 0.61), and phosphate carrier (*FCC* = 0.60). The highest control was exerted by adenine nucleotide translocase (ANT), *FCC* = 1.02 (Kaambre et al., 2012). Our preliminary data for HCC showed high FCCs for: Complex-I (*FCC* = 0.62), Complex-III (*FCC* = 0.73), and Complex-IV (*FCC* = 0.58).

**Figure 1:**
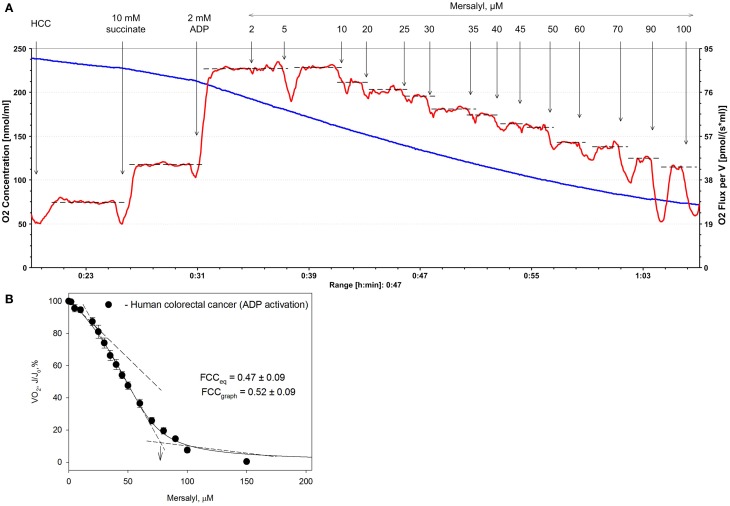
**Representative traces of change in the rate of oxygen consumption by permeabilized human colorectal cancer (HCC) fibers after their titration with increasing concentrations of mersalyl, an inhibitor of inorganic phosphate carrier (panel A)**. The values of respiration rate obtained were plotted vs. mersalyl concentration (panel **B**) and from the plot the corresponding flux control coefficient was calculated. Bars are ±SEM.

The FCC was calculated graphically as shown in Figure 1 and as explained in Methods. In the case of HCC, the FCC-values for PIC was calculated as 0.52 (graphic method) (Groen et al., 1982) and 0.47 [according to the Small equation (Small, 1993)]. Although both these methods gave similar values, the use of the Small equation for calculating of FCC(s) was preferred over the graphical one because it is more robust and less subjective. Further investigations are needed to determine the FCC-values for other respiratory chain complexes in HCC and in healthy colon tissue.

In the case of HBC, the summation of the determined FCC(s) for all steps evaluated in the ATP synthasome and ETC complexes was found to be around 4. This value significantly exceeds the theoretically expected summation for linear pathways (value 1). According to Lenaz et al. (2010), a sum of FCC(s) exceeding 1 indicates the existence of supramolecular association of the respiratory complexes that was confirmed by electron microscopy, native gel electrophoresis and single particle image processing (Lenaz and Genova, 2009, 2010). Although more studies are needed to elucidate this important matter, supercomplex formation would allow to explain, at least in part, the high intrinsic resistance to apoptotic stimuli that tumor cells exhibit, namely via suppression of cytochrome-c release. The formation of respiratory supercomplexes could occur not only in HBC but also in HCC cells.

## Conclusion

In this work we show that MCA can be applied to *in situ* quantitative analysis of respiration in cancerous and normal tissues obtained from small amounts of biopsy material. *In situ* studies have the advantage of preserving the cellular ultrastructure such as the cytoskeleton thus enabling the study of their role in controlling energetics in cancer cells. It is important to emphasize that the use of MCA for studying mitochondrial function *in situ* allows us to avoid changes in microenvironment that happen during the isolation procedure. Our studies were performed on cells from tissue samples isolated from patients. This may represent a limitation because recently it has been emphasized that the bioenergetic profile of tumors cells depends largely, among other factors, on the stage of tumor growth and its degree of vascularization (Moreno-Sanchez et al., 2007). Large number of studies were performed on tumor cell cultures, which exhibit a strong dependency on glycolysis, but there might be a strong impact of the artificial culture conditions on energy metabolism (Jose and Rossignol, 2013). One example of the impact of cell culture, the so-called “culture shock,” modulates the activity of some genes, which possibly upregulate glycolysis (Gnaiger and Kemp, 1990; Gstraunthaler et al., 1999). This specificity underscores the importance of examining tumor cell behavior in their natural environment.

We quantified the control of respiration in two different types of human cancer cells. The main result obtained is that the ANT exerts a high flux control, implicating the role of adenine nucleotide exchange between mitochondrial and cytoplasmic compartments as a key energetic trait in cancer cells. This result may be important for cancer therapy. Possible suppression of ANT2 and/or overexpression of ANT1 and ANT3 isoforms in cancer cells may induce their death via apoptosis (Jang et al., 2008). We also show that HCC cells exhibit increased respiratory rates as compared to adjacent normal cells suggesting the presence of “reverse Warburg” effect (Whitaker-Menezes et al., 2011). The novel concept of reverse Warburg in cancer metabolism denotes that tumor cells provoke aerobic glycolysis in the tumor stroma thus lactate secretion from cancer-associated fibroblasts. Secreted lactate then fuels OXPHOS in epithelial cancer cells, by acting as a paracrine onco-metabolite. Our data suggest a new strategy for HCC treatment; namely, by inhibitors of some monocarboxylate transporters (Queiros et al., 2012).

From our MCA studies it can be inferred the presence of respiratory supercomplexes in mitochondria from cancer cells. Recent investigations have shown that respiratory chain complexes I, III and IV can interact to form supercomplexes (respirasomes) (Acín-Pérez et al., 2008; Lenaz and Genova, 2010; Dudkina et al., 2011). Future studies using MCA should unravel how the FCC-value depend upon structural organization of the respirasomes and how exactly the respiratory chain is organized in tumor cells.

Further development in the area of metabolic flux analysis and cellular bioenergetics is important to link future studies on tumor metabolomics to clinical research.

### Conflict of interest statement

The authors declare that the research was conducted in the absence of any commercial or financial relationships that could be construed as a potential conflict of interest.
